# B cell Semaphorin 4c expression mitigates the airway hyperresponsiveness and acute inflammation which characterize allergic airway disease

**DOI:** 10.1186/1710-1492-10-S2-A26

**Published:** 2014-12-18

**Authors:** Alex Lei, Di Xue, Marylin Desjardins, Marianne Beland, Bruce Mazer

**Affiliations:** 1 Meakins-Christie Laboratories, Department of Experimental Medicine, McGill University, Montreal, Quebec, H2X 2P2, Canada; 2 Montreal Children’s Hospital, Department of Pediatrics, McGill University Health Center, Montreal, Quebec, H3H 1P3, Canada

## Background

Semaphorin signaling proteins, initially examined in the context of neuronal axon development, have recently been implicated as regulators of immune cell migration. Our laboratory has determined that expression of Semaphorin 4C (Sema4C) is strongly induced on B cells exposed to Th2 stimulation, and we seek to elucidate its mechanism of controlling allergic airway disease.

## Methods

Wild-type and Sema4C^-/-^ mice were sensitized intraperitoneally using 100 μL OVA (0.5 mg/mL ovalbumin and 4 mg/mL aluminum hydroxide in PBS) on days 0 and 14, and were challenged intranasally using 20 μL OVA (10 mg/mL ovalbumin in PBS) from days 28 to 30. Sacrifice and analysis of Airway Hyperresponsiveness via flexiVent was performed on day 31. Serum IgE and IL-10 expression levels were measured by ELISA. B cells were phenotyped by fluorescence-activated cell sorting (FACS). B cell motility was measured by migration assays.

## Results

**Figure 1:**
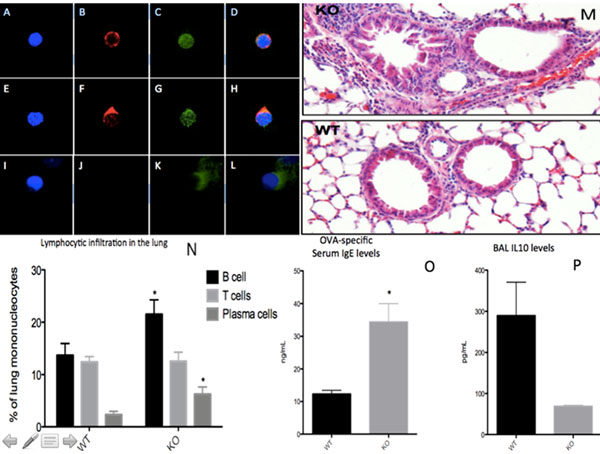
**A-H.** Sema4C localized at B cellular synapses for wild-type mice (WT). Immunofluorescence staining of B cells stimulated with CD40L, IL-4, and IL-21 for 24 hours (A-D), or 72 hours (E-H). **I-L.** Sema4C^-/-^ (KO) B cells have aberrant actin cytoskeletal structural organization. **M.** KO mice have narrower airway diameter than WT mice. H&E staining of WT (n=6) and KO (n=5) lungs with Allergic Airway Disease (AAD). **N**. KO mice with AAD had a greater percentage of lung-infiltrating lymphocytes (CD4+ T cells, CD19+ B cells, and CD138+ plasma cells) than WT mice. Lymphocytes were recovered using Bronchoalveolar Lavage. **E.** KO mice with AAD had higher serum OVA-specific IgE levels than WT mice. **F.** KO mice with AAD had lower serum IL-10 levels than WT mice.

Please see figure 1.

## Conclusions

Semaphorin 4C regulates the allergic airway disease through immune synapse-governed cytoskeletal rearrangements in B cells, and minimizes the inflammatory cellular lung infiltration that contributes to airway hyperresponsiveness.

